# Bacterial Outer Membrane Vesicles as a Versatile Tool in Vaccine Research and the Fight against Antimicrobial Resistance

**DOI:** 10.1128/mBio.01707-21

**Published:** 2021-08-10

**Authors:** Zhuang Zhu, Fabio Antenucci, Kasper Rømer Villumsen, Anders Miki Bojesen

**Affiliations:** a Department of Veterinary and Animal Sciences, Faculty of Health and Medical Sciences, University of Copenhagen, Frederiksberg, Denmark; University of Texas Health Science Center at Houston

**Keywords:** antimicrobial resistance, bacterial outer membrane vesicles, vaccine

## Abstract

Gram-negative bacteria include a number of pathogens that cause disease in humans and animals. Although antibiotics are still effective in treating a considerable range of infections caused by Gram-negative bacteria, the alarming increase of antimicrobial resistance (AMR) induced by excessive use of antibiotics has raised global concerns. Therefore, alternative strategies must be developed to prevent and treat bacterial infections and prevent the advent of a postantibiotic era. Vaccines, one of the greatest achievements in the history of medical science, hold extraordinary potential to prevent bacterial infections and thereby reduce the need for antibiotics. Novel bacterial vaccines are urgently needed, however, and outer membrane vesicles (OMVs), naturally produced by Gram-negative bacteria, represent a promising and versatile tool that can be employed as adjuvants, antigens, and delivery platforms in the development of vaccines against Gram-negative bacteria. Here, we provide an overview of the many roles OMVs can play in vaccine development and the mechanisms behind these applications. Methods to improve OMV yields and a comparison of different strategies for OMV isolation aiming at cost-effective production of OMV-based vaccines are also reviewed.

## INTRODUCTION

### Antimicrobial resistance.

Human beings have greatly benefited from the discovery and application of antibiotics to fight bacterial infections and significantly improve health and life span as well as enable efficient animal husbandry and food production (1, 2). For decades, however, antibiotics have been used in an unrestrained manner, resulting in the global emergence of antimicrobial resistance (AMR). The World Health Organization (WHO) has warned that the world is at the brink of a postantibiotic era (3), where a reduced effectiveness of existing antibiotics and lack of novel antibiotics will represent major threats to the health of humans and animals. The problem is particularly pressing in cases of infections caused by Gram-negative bacteria, since no new antibiotic classes active against Gram-negative bacteria have been approved in the past 20 years (4, 5). This alarming situation has been largely attributed to the imprudent and excessive use of antibiotics in humans and animals (6). Of particular concern is antimicrobial use in the livestock industry, which is a major contributor to the rapidly increasing antimicrobial consumption due to a large global expansion of livestock production reliant on routine antibiotic use to maintain animal health and productivity (2). The global antimicrobial consumption in food animals is expected to rise by 67% from 2010 to 2030, owing to the rising protein demand, especially in middle-income countries (7, 8). Particularly concerning is the overlap in the spectra of antimicrobials used in humans and animals, exemplifying how the misuse and overuse of antimicrobials in food animals can accelerate the emergence of antimicrobial-resistant bacteria, threatening the health of both humans and animals (9). The public crisis elicited by AMR pinpoints the interdependent health relationship between human and animals and calls for global attention to address the problem from a One Health perspective (10, 11). This has led to the adoption of numerous measures worldwide to limit the use of antimicrobials in food animals, including, for example, bans on antimicrobial growth promoters and restrictions on the use of therapeutic antimicrobials critically important in human medicine. Unfortunately, these measures have been only partly effective in reducing antimicrobial use, and the surge of AMR in medically relevant bacterial species remains a serious health threat (12).

### Vaccines and other alternatives to antimicrobials.

To prevent selection of antimicrobial-resistant bacterial strains, strategies that aim at reducing antimicrobial use through a One Health approach, targeting both humans and animals, are needed (13). Several interventions may contribute to mitigate the development of AMR, including improved hygiene and sanitation, better management and husbandry practices, vaccination, and novel technologies, like phage therapy and monoclonal antibody therapy (14, 15). Among these alternatives, vaccination is arguably one of the most effective in reducing the need for antimicrobial treatment. This is exemplified by the vaccines developed against Streptococcus pneumoniae and Haemophilus influenzae type b, which dramatically reduced antibiotic use in humans (16, 17), and the development and application of vaccines that, together with proper management, significantly limited the use of antibiotics in aquaculture in Northern Europe and North America (18). Vaccines have been regarded as optimal tools in the fight against bacterial infections thanks to their ability to elicit specific immunity in vaccinated individuals (19), consequently reducing antibiotic use directly. In addition, even vaccines against nonbacterial pathogens, such as viruses, can indirectly limit antibiotic use by preventing secondary bacterial infections frequently occurring as a result of viral infections (20). In light of the proven potential of vaccines in combating AMR, focus on research, including development of effective adjuvants, identification of novel vaccine candidates, and vaccine delivery systems, is warranted.

### Antigenic variation in bacteria.

A major challenge associated with bacterial vaccine design is the vast antigenic diversity displayed by the great majority of bacteria (21). Bacteria are able to alter their antigenic profiles as part of a host immune evasion strategy, challenging development of broadly protective vaccines (22). Even within the same species, antigenic specificity can vary significantly between different strains. This is exemplified by Neisseria meningitidis, in which 13 serogroups have been proposed based on the capsular polysaccharides, six of which are responsible for the great majority of clinical cases (23). Development of a multivalent and universal vaccine against N. meningitidis has been hampered by the antigenic diversity present between different serogroups. However, the problems posed by antigenic diversity are not limited to a cross-protective vaccine, as exemplified by the monovalent vaccine against N. meningitidis serogroup B (MenB), for which the effectiveness has been limited by the antigenic variation of outer membrane proteins used as protective antigens in the vaccine formulation employed (24, 25). Therefore, a good, rational vaccine design requires identification of sufficiently conserved antigens with excellent protective capacity. Bacterial outer membrane vesicles (OMVs) largely encompass these requirements, which we will address in detail in the following sections.

## OMVS AND INTERACTIONS WITH IMMUNE SYSTEM

### OMVs.

**FIG 1:**
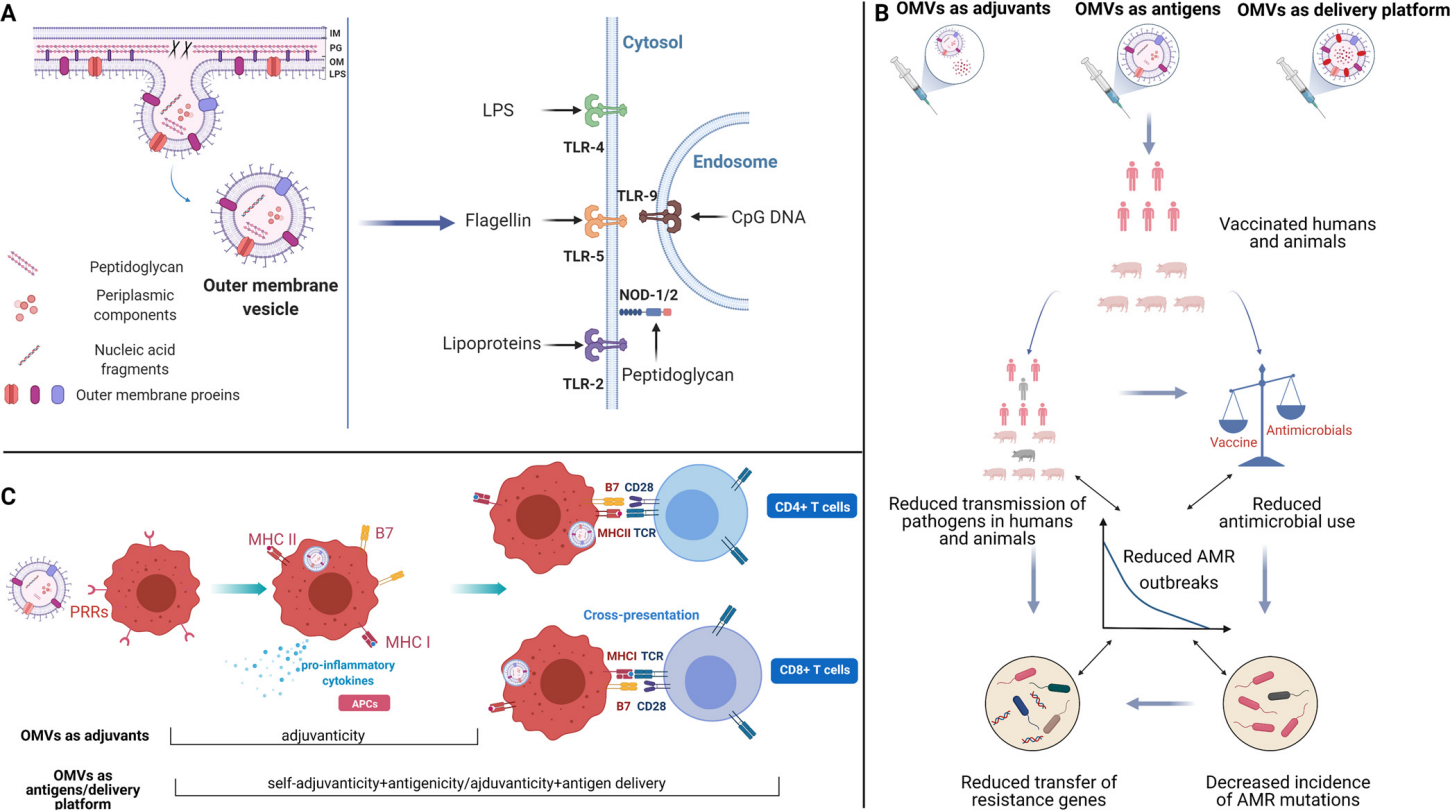
(A) Biosynthesis of outer membrane vesicles (OMVs) and recognition of OMV-associated pathogen-associated molecular patterns (PAMPs) by pattern recognition receptors (PRRs). Arrows indicate specific ligand-receptor interactions between PAMPs and Toll-like receptors (TLRs) or NOD-like receptors (NLRs). IM, inner membrane; PG, peptidoglycan; OM, outer membrane; LPS, lipopolysaccharide. (B) Schematic representation of pathways by which OMVs can contribute to reducing the insurgence of antimicrobial resistance (AMR). (C) Schematic representation of OMV-associated antigens processing and presentation to T cells. APCs, antigen-presenting cells; PRRs, pattern recognition receptors; MHC, major histocompatibility complex; TCR, T-cell receptor; CD, cluster of differentiation. Created with BioRender.com.

Outer membrane vesicles (OMVs) are spherical particles secreted by all Gram-negative bacteria investigated to date (26). Although the existence of OMVs has been known to scientists for decades, the mechanisms involved in OMV formation remains elusive at present (27). In principle, the formation of OMVs starts from the breakage of links between the bacterial outer membrane and underlying peptidoglycan (PG) layer. Affected regions of the outer membrane then protrude to form vesicular buds, which continuously bulge outwards until detaching from the remaining outer membrane, and give rise to OMVs (Fig. 1A) (28). As protrusions of the parental cell’s outer membrane, OMVs inherit a composition similar to that of the outer membrane, consisting of lipopolysaccaride (LPS), outer membrane proteins (OMPs), and PG. This essentially makes them small, non-live antigenic representations of the parent cell, including the surface complexity, in a way that vaccines made from recombinant proteins cannot. Additionally, OMVs have been found to contain periplasmic components selected by an unclear sorting mechanism (Fig. 1A) (29). These molecules are preserved in native conformation, further highlighting OMVs’ potential for vaccine development. The structure of OMVs and enclosed molecules make them accessible and immunostimulatory to immune cells, endowing OMVs with inherent adjuvanticity and immunogenicity (see “Interactions between OMVs and the immune system,” below). Meanwhile, ease of manipulation makes OMVs suitable antigen delivery platforms (30). Multifaceted functions of OMVs as adjuvants, antigens, and delivery platforms in vaccine research have been demonstrated, showing their potential as contributors to reduced bacterial infection and AMR (Fig. 1B).

### Interactions between OMVs and the immune system.

The host innate immune system can recognize OMVs efficiently, facilitating induction of inflammatory response and antigen presentation and subsequently leading to a specific immune response to OMVs. The initiation of this process is largely attributed to the presence of pathogen-associated molecular patterns (PAMPs) on the OMVs. PAMPs are molecules, such as LPS or PG, that are widely conserved among microorganisms. PAMPs present on the OMVs can be detected by the pattern recognition receptors (PRRs) ubiquitously expressed by innate immune cells that, in turn, initiate signal cascades, leading to the priming of the host immune response (Fig. 1A). This can induce adaptive immunity against either OMV-associated or coadministered antigens.

### (i) Inflammatory response.

The interplay between OMV-associated PAMPs and innate immune cells plays an important role through induction of proinflammatory cytokine secretion by different cell types (31–33). Among the important PRRs, Toll-like receptors (TLRs) are capable of detecting the PAMPs present on the OMVs (Fig. 1A) (34). LPS, flagellin, and lipoproteins are ligands of surface receptors TLR4, TLR5, and TLR2, respectively. One of the intracellular TLRs of major interest is TLR9, responsible for the recognition of CpG DNA, which are cytosine-phosphate-guanine dideoxynucleotide-rich motifs commonly found in bacterial and viral genomes. Receptors other than TLRs also participate in the recognition of OMV-associated PAMPs. For example, OMV-associated LPS has access to the cytosol of immune cells through endocytosis and is sensed by caspase-11, inducing cell death and interleukin-1 (IL-1) responses during bacterial infections (35). Likewise, OMVs deliver PG to cytosolic NOD-like receptors and contribute to induction of inflammatory responses (36, 37). Damaged cells induced by OMVs also can release damage-associated molecular patterns as danger signals to promote inflammatory responses. Accordingly, crucial proinflammatory cytokines are released as a result of interactions between OMV-associated PAMPs and innate immune cell signals, contributing to key responses against bacterial infection, including recruitment and activation of additional immune cells, phagocytosis of bacterial cells, and initiation of adaptive immunity.

### (ii) Antigen presentation.

The interactions between PAMPs and innate PRRs promote activation and maturation of antigen-presenting cells (APCs) in the transition toward adaptive immune responses. Activated APCs can express a variety of surface molecules that are needed for presentation of protein antigens to T cells. As shown in Fig. 1C, APCs stimulated by OMVs express major histocompatibility complex class II (MHC-II) as well as B7 proteins, which are necessary costimulators for T cell activation. Internalized OMVs are processed by APCs, in which protein antigens from OMVs are processed and loaded onto MHC-II for presentation to T cell receptors as signal I in T cell activation. Recognition of B7 costimulators through their ligands induces signal II; together, these two signals provide the stimulation needed for CD4^+^ T cell activation.

Besides presentation to CD4^+^ T cells, it is worth noting that OMVs, as a mixture of exogenous antigens, are capable of programming APCs for antigen presentation to CD8^+^ T cells (referred to as cross-presentation). Lee et al. showed that OMVs could enhance cross-priming of CD8^+^ T cells when coimmunized with antigens as adjuvants, indicated by increased gamma interferon (IFN-γ) secretion of mice splenocytes restimulated with MHC-I-restricted peptides (38). Moreover, it has been shown that engineered OMVs displaying specific antigens induced maturation of mouse and human dendritic cells and programmed dendritic cells for antigen-specific cross-presentation via the MyD88 signaling pathway (39). This study also hypothesized that antigens displayed on the surface of OMVs are more inclined to be presented to CD8^+^ T cells, whereas antigens present in the lumen of OMVs were shown mainly to induce a CD4^+^ T cell response while failing to induce a CD8^+^ T cell response. Taken together, these studies indicate that the localization of OMV-associated antigens plays a role in determining which immune effectors and pathways are activated following OMV administration. The mechanism behind the cytosolic localization of OMV-associated protein antigens, which is necessary for the occurrence of cross-presentation, remains unknown. Nevertheless, the cross-presentation-inducing ability is of great importance for the utilization of OMVs as therapeutics in treatments that rely on the activation of CD8^+^ T cells, such as treatments against viral infection and tumors.

## OUTER MEMBRANE VESICLES IN VACCINE DEVELOPMENT

### OMVs as adjuvants.

The main concept of modern vaccine design is to replicate the activation of the host immune response induced by natural infections while avoiding the morbidity associated with it (40). Earlier vaccine designs have largely relied on attenuated or inactivated pathogens due to a combination of technical limitations and ease of development and production of such vaccines. More recently, the elucidation of some of the principles behind microbial pathogenesis and immune response have allowed the identification and isolation of antigens that are essential for inducing protective immunity toward pathogens. These antigens can then be dissected and purified for the development of new vaccines with a comparatively higher safety profile compared to whole-cell-killed vaccines or live attenuated vaccines. However, highly purified antigens are often devoid of adjuvant properties, and vaccines based on purified antigens alone may present reduced immunogenicity and, thus, be less effective (41). The limited immunogenicity of vaccine formulations based on purified antigens can be complemented by coadministration of exogenous adjuvants, which are immune potentiators enhancing the immune response. Generally, adjuvants can be categorized into two major classes according to the mode of action: (i) direct activation or enhancement of the host immune response and (ii) enhancement of antigen delivery by facilitating uptake, transport, or presentation of antigens by APCs (41). Several mechanisms have been proposed to explain how different adjuvants exert their effect (42). One example of the first class of adjuvants is offered by the monophosphorylated lipid A, a licensed adjuvant consisting of a mixture of monophosphorylated lipids, which mimics the structure of lipid A in LPS, acting as a PAMP to stimulate the immune response through PRRs. An example of the second class of adjuvants is instead the depot effect exerted by aluminum hydroxide, which can absorb antigens and then slowly release them, prolonging the duration of the antigen exposure and immune response after administration of the vaccine formulation.

Originating from bacterial membranes, OMVs display a complex array of PAMPs, such as LPS, flagellin, and PG, in their native conformation. These molecules are capable of triggering the innate immune response by inducing an increased expression of surface molecules and release of proinflammatory cytokines in innate immune cells. PAMPs in OMVs from various bacteria have been demonstrated to be able to stimulate the innate immune response in many cell types, as summarized in Table 1. Following activation, innate effectors interact with adaptive effectors to elicit a multifaceted immune response against specific antigens, as shown in Fig. 1C. Additionally, OMVs have been shown in some instances to carry virulence factors involved in host cell damage, which could trigger localized release of danger signals that would, in turn, stimulate immune and inflammatory responses. Finally, it has been proposed that OMVs influence the immune response by what is defined as the “geographic concept,” namely, ensuring the spatial availability of antigens to immune effectors. OMVs could play a role in this by increasing antigen uptake by APCs and subsequent efficient translocation of OMVs and associated antigens to lymph nodes (43, 44).

**TABLE 1:** OMV-associated PAMPs contributing to innate immune response in various cell types

Content	Species	Cell model	Relevant result(s)	Reference
LPS	Pseudomonas aeruginosa	Mouse macrophages	Both LPS and protein components on OMVs from Pseudomonas aeruginosa were responsible for eliciting innate immune response	32
	Neisseria meningitidis	Human monomacrophages, human dendritic cells	Neisseria meningitidis OMVs with less LPS or less toxic LPS induced weaker cytokine response	85
	Escherichia coli	Human macrophages	LPS-neutralized OMVs induced weaker inflammatory response	113
OMPs	Pseudomonas aeruginosa	Mouse macrophages	Both LPS and protein components on OMVs from Pseudomonas aeruginosa were responsible for eliciting innate immune response	32
	Acinetobacter baumannii	Human laryngeal epithelial cells	Surface-exposed membrane proteins in Acinetobacter baumannii OMVs induced proinflamatory cytokine response	114
Flagellin	Enterohemorrhagic Escherichia coli	Human intestinal epithelial cells	H7 flagellin was the key IL-8-inducing component of EHEC O157:H7 OMVs	115
	Pseudomonas aeruginosa	Rat renal epithelial cells	Both LPS and protein components on OMVs from Pseudomonas aeruginosa are responsible for eliciting innate immune response	32
Peptidoglycan	Helicobacter pylori, Pseudomonas aeruginosa, Neisseria gonorrhoeae	Human epithelial cells	Bacterial OMVs delivered peptidoglycan to cytosolic NOD-1 and activated NF-kB pathway	116
	Aggregatibacter actinomycetemcomitans	Human gingival fibroblasts	Internalized OMVs from Aggregatibacter actinomycetemcomitans delivered peptidoglycan to NOD-2 receptors	37
CpG DNA	Moraxella catarrhalis	Human tonsillar B cells	DNA associated with OMV induced full B cell activation by signaling through TLR9	117

In summary, a complex adjuvant effect can be attributed to the mix of PAMPs on the OMV surface, introduction of danger molecules as a consequence of OMV cytotoxicity, and an effect on antigen availability and efficient presentation to the immune effector cells (geographical concept).

### OMVs as antigens.

OMVs can be viewed as nonreplicative representations of the parental bacterial cell by possessing antigenicity similar to and a higher safety profile than intact yet attenuated bacterial cells. In addition to PAMPs, OMVs also carry a combination of pathogen-specific antigens that can stimulate long-lasting adaptive immunity and immune memory, both of which are crucial in conferring protection against future infections. Furthermore, the inactivation procedure during production of conventional bacterial vaccines or subunit vaccines typically denatures proteins and other components, potentially reducing the antigenic diversity in these vaccine types. In comparison, OMV-based vaccines offer the potential of providing a broader and more effective protection by presenting a repertoire of antigens in their native conformation. Thus, OMVs are regarded as promising vaccine candidates against the bacteria from which they are derived. A good example is the recent OMV-based vaccine against the meningococcus MenB (45). Several vaccines containing OMVs against MenB have, in fact, been independently developed and approved for use in the past few decades (46–48), yet the vaccine known as 4CMenB (Bexsero; GSK) reached a milestone of being licensed in more than 35 countries (49, 50), which further highlights the potential of OMVs for vaccine development. Together with three recombinant proteins, the OMVs in 4CMenB confer broad protection against a wide variety of antigenically different MenB strains (50). The success of MenB vaccines has inspired extensive research into the potential of OMVs in the development of novel vaccines against human and animal bacterial pathogens. OMVs have been administered alone or in combination with purified antigens and tested on appropriate animal models, as summarized in Table S1 in the supplemental material. A major safety concern using OMVs in vaccine formulations is their LPS content stemming from their complex, natural compositions, as this may lead to adverse effects, including septic shock and death in humans and some animal species (51). However, the response to LPS appears to be dose dependent and mostly limited to mammalian species, as some animals, such as chicken and fish, appear to be quite insensitive to LPS (52). Therefore, it would be advisable to assess whether detoxification of LPS is necessary on the basis of which host species the OMV vaccine is developed for. When necessary, LPS can be detoxified by manipulating genes involved in the acylation or phosphorylation of lipid A to produce modified LPS, such as penta-acylated lipid A and monophosphoryl lipid A. OMVs prepared from bacteria with modified LPS retain adjuvanticity but have low toxicity (53).

10.1128/mBio.01707-21.1 TABLE S1 Examples of experimental OMV-based vaccines. Download TABLE S1, DOCX file, 0.04 MB. Copyright © 2021 Zhu et al. 2021 Zhu et al. https://creativecommons.org/licenses/by/4.0/ This content is distributed under the terms of the Creative Commons Attribution 4.0 International license.

### OMVs as antigen-delivering platforms.

Although it is theoretically possible to develop vaccines against all species of Gram-negative bacteria with homologous OMVs, as nearly all Gram-negative bacteria release OMVs both *in vitro* and *in vivo*, not all Gram-negative bacteria are equally suitable for OMV isolation. This can be due to strict growth requirements, low growth rate, low vesiculation rate, or a combination of these factors, which render the OMV yield too low (54). In this context, OMVs from high-producing and well-characterized species can be developed as universal platforms to deliver homologous and heterologous antigens.

One of the most important factors that affect vaccine immunogenicity is antigen size. Antigens in the 10 nm to 5 μm range are preferably transported to lymphoid organs, leading to enhanced uptake into APCs, as exemplified by an early study that showed that microspheres larger than 5 μm were not efficiently transported to the spleen (55). In vaccine design, delivery systems such as nanoparticles, microparticles, and various emulsions are commonly used in the formulation to form larger particles loaded with antigens (56). OMVs vary in a range of 20 to 300 nm in diameter, a size that allows OMVs to enter the lymphatic vessels efficiently and reach lymphoid organs directly without the need for carriers (57, 58). As discussed in the following section, several studies have explored the potential of using OMVs to deliver different types of antigens.

### (i) Protein antigen delivery.

OMVs can be enriched with both homologous and heterologous cargo of bacterial, viral, or other origin, through either membrane enrichment or luminal packaging (59–61). Loading proteins to the OMV surface can be achieved in a few ways, including carrier proteins such as outer membrane proteins, transmembrane proteins, or autotransporters. One of the best-described carrier proteins, ClyA, a cytolysin secreted along with the release of OMVs, has been widely exploited as a fusion partner for surface display of protein antigens (62, 63). Antigens fused to ClyA present the additional benefit of enhanced immunogenicity, as ClyA is capable of eliciting a stronger immune response (64).

Another class of antigen carriers is represented by autotransporters, such as hemoglobin protease (Hbp). Hbp consists of a cleavable β-domain that anchors on the outer membrane and a passenger domain that can be replaced with heterologous antigens of interest. Normally, Hbp synthesized in bacteria is translocated across the cell envelope and secreted into the extracellular milieu by the enzymatic lysis of the link between passenger and β-domain. To achieve the purpose of surface display, a noncleavable mutation was introduced into Hbp to keep heterologous passengers retained on the outer membrane. Based on this principle, the feasibility of employing Hbp for heterologous protein display in OMVs has been demonstrated and applied in experimental vaccine research (65–67). Alternatively, a semisynthetic way of combining Hbp carrier with a Tag/Catcher protein ligation system has been developed to couple purified proteins to Hbp-displaying OMVs (68, 69). This strategy extends the applicability of Hbp to allow surface enrichment of OMVs with large or multiple proteins of an external source.

It is worth mentioning that most of the studies related to OMV antigen display have been conducted in Escherichia coli and Salmonella spp. The potential of alternative carrier proteins expressed by less well-characterized bacterial species still remains to be investigated. Some examples of alternative carrier systems are the proteins fHbp and ApfA, adapted for membrane enrichment of antigens in N. meningitidis and Actinobacillus pleuropneumoniae, respectively (70, 71).

Proteins can also be incorporated into the lumen of OMVs by fusion to either secretion signals like the twin-arginine (Tat) signal sequence or to periplasmic proteins such as OmpA (72, 73). However, it is controversial whether luminal antigens are as effective as surface antigens in terms of stimulating an immune response, as their localization prevents direct access by immune effectors. In some studies, only minor specific antibody responses were induced by luminally placed antigens (59, 74). In contrast, Fantappie et al. showed that luminal antigens were able to induce high antibody titers with excellent functional activity and proposed that the native conformation of luminal antigens appeared to be an essential factor for eliciting an effective antibody response (75). Therefore, the position of the protein antigens may affect the protective efficacy in an antigen-specific manner.

### (ii) Glycan antigen delivery.

Glycan antigens exist in all domains of life and represent an important group of specific antigens able to confer protective immunity. This is particularly true in the case of pathogens that are highly susceptible to the targeting of capsular polysaccharide by immune effectors, such as S. pneumoniae and Campylobacter jejuni (76).

When administered alone, glycans usually fail to induce long-lasting immune responses and T cell memory (77). Therefore, vaccines based on glycan antigens are often conjugates consisting of protein carriers and glycan antigens. Although both chemical and biosynthetic coupling technologies are available to develop glycoconjugate vaccines, they still offer low yield, high complexity of production, and a general lack of versatility posed by the substrate specificity of glycosyltransferase and limited glycosylation sites of the protein carriers (78, 79). In that context, OMVs have been exploited as an alternative way to deliver glycan antigens.

As a proof of concept, Chen et al. first described that the heterologous O-antigen polysaccharide from Francisella tularensis could be incorporated into E. coli OMVs, which lack their own O-antigen polysaccharide. Mice immunized with the resulting recombinant glycan-enriched OMVs showed significantly longer survival time after lethal F. tularensis challenge (53). Subsequent studies incorporated OMVs displaying a variety of glycans. Price et al. employed E. coli OMVs as a platform to deliver S. pneumoniae capsule polysaccharide and was able to induce a specific antibody response that was comparable to the one induced by the commercial vaccine Prevnar 13 (76). Stevenson et al. (80) reported poly-N-acetyl-d-glucosamine (known as PNAG), displayed by E. coli OMVs able to elicit PNAG-specific antibodies. However, antibodies against PNAG in its native acetylated conformation were shown to be ineffective in preventing infection from PNAG-positive bacteria (81). To address this, OMVs displaying deacetylated PNAG (dPNAG) were developed. Antibodies against dPNAG were capable of mediating *in vitro* killing of different species of bacteria (80).

Collectively, OMVs seem to enable glycan antigens to stimulate an immune response effectively like protein carriers do and, at the same time, can circumvent some drawbacks affecting other antigen delivery mechanisms. Thanks to their adjuvant properties, size, and versatility, OMVs represent efficient carriers for the delivery of various glycans from a broad range of pathogens.

## METHODS FOR INCREASING OMV YIELDS AND EFFECTIVE ISOLATION

Vaccines targeted for human and livestock use require large-scale production capacity in order to fulfill market demands. Traditional OMV-based vaccines are challenged by low yields and complex isolation procedures, which often result in high production costs that are untenable, particularly in the animal heath market. To pave the way for the development of more OMV-based vaccines, novel and cost-effective protocols to increase OMV yields need to be implemented (30).

### Increasing OMV yields.

Under normal conditions, OMV secretion is mostly regulated by growth phases, which influence OMV production both quantitatively and qualitatively (82). Therefore, an optimal harvest time, generally late exponential phase in most cases, is required to obtain as high a yield of OMVs as possible while avoiding contaminations induced by bacterial cell lysis after long cultivation (83). On this basis, additional methods for increasing OMV yields, including physical, chemical, and genetic techniques, have been investigated as listed in Table S2.

10.1128/mBio.01707-21.2 TABLE S2 Methods for increasing OMV yields. Download TABLE S2, DOCX file, 0.03 MB. Copyright © 2021 Zhu et al. 2021 Zhu et al. https://creativecommons.org/licenses/by/4.0/ This content is distributed under the terms of the Creative Commons Attribution 4.0 International license.

Sonication is a common physical method employed for OMV production, resulting in the disruption of bacterial cells into particles which then assemble into artificial OMVs. One of the main issues associated with this methodology is that the composition of the OMVs produced by sonication may differ greatly from that of naturally released OMVs. For instance, cytoplasmic proteins have been found to predominate in protein profiles from OMVs artificially produced from *Glaesserella parasuis*, while proteins from the outer membrane and periplasm dominated in naturally released OMVs (84). Temperature shift is another physical method that may influence OMV secretion in a species-specific manner. Both high- and low-temperature conditions have been shown to increase OMV production depending on the species (82).

OMV secretion may also be regulated by the addition of specific organic or inorganic chemicals to bacterial cultures, either stimulating bacteria directly to increase vesiculation or inducing environmental stress, which results in increased vesiculation. One such method is detergent-based extraction, a well-characterized technique utilized in the preparation of OMVs from N. meningitidis. One of the advantages of detergent extraction is that this method not only results in increased OMV yields but also reduces LPS content in the isolated OMVs (85). Metal chelators represent another class of molecules capable of increasing OMV yields by depleting metal ions in the solution, creating restricting growth conditions that often result in increased bacterial vesiculation. Finally, environmental stress such as oxidative stress can be induced by adding hydrogen peroxide or depleting cysteine, triggering survival responses and hypervesiculation in certain bacterial species (86, 87).

Gene knockout is a well-established method for selective inactivation of specific genes. This method can be employed for engineering OMV yields as well by targeting key genes involved directly or indirectly in OMV biogenesis. Manipulation of genes affecting the cross-links between the outer membrane and the PG layer, PG degradation, and membrane curvature represents examples that may lead to increased blebbing of the outer membrane and increased OMV secretion in the mutants. OMVs generated by genetically modified hypervesiculating bacteria are also referred to as generalized modules for membrane antigens (GMMA) and are regarded as a practical source of membrane antigens in vaccine manufacturing (88, 89).

The methods discussed in this section are efficient ways of increasing the limit imposed by low OMV yields under usual culturing conditions. The resulting OMV isolates vary in composition and can be categorized into various types depending on the method used for increasing yields (Table S2). In industrial vaccine production, methods for scaling up OMV yields are needed to maximize the volume produced and minimize the production costs. Gerritzen et al. demonstrated that in N. meningitidis, shifting the OMV production mode from batch to continuous process resulted in an estimated 9-fold increase of OMV production under a specific dilution rate (90). This shift in production methodology improved productivity without compromising the overall properties of the isolated OMVs. A similar strategy may represent the next step for increasing OMV yields in other bacterial species.

### OMV isolation efficiency.

The majority of protocols for OMV isolation start with one or more low-speed centrifugation steps, followed by the subsequent filtration of supernatants in order to produce cell-free filtrates (82, 91). The resulting filtrate is then further processed, concentrated, and purified via a range of techniques, comparatively arranged in terms of advantages and disadvantages in Table 2.

**TABLE 2:** Comparison of methods for isolating OMVs

Method	Advantage	Disadvantage	Reference(s)
Differential centrifugation	Low technical requirements; ease of execution	Laborious, low purity, generally needs to be combined with density gradient centrifugation for further purification	118
Size-exclusion chromatography	Rapid isolation process; high purity	High cost; unsuitable for large-scale production	94
Hydrostatic filtration dialysis	Low cost; suitable for large scale production	Limited data on purity of the isolated OMVs	95, 98
Affinity purification	Fast; specific isolation of targeted OMV populations	Only available for OMVs carrying exposed tags; low recovery rate	99

Differential centrifugation (DC) is the most widely used method for OMV isolation. This technique consists of multiple steps of low-speed centrifugation followed by a final ultracentrifugation step, which ultimately leads to the production of an OMV pellet. Although simplicity of execution and relatively low technical requirements characterize DC as one of the most approachable methods for OMV isolation, contaminants such as pili, flagella, and soluble components, which cannot be separated by centrifugation alone, are still present in the isolated OMV pellet (92). Therefore, OMVs isolated by DC usually requires additional separation steps, such as density gradient centrifugation, that relies on either iodixanol or sucrose as the separation medium to produce high-purity OMVs (26). Moreover, ultracentrifugation is a relatively lengthy and laborious process due to the inability to process large sample volumes. Repeated centrifugation or extra preconcentration steps are required when large volumes of samples need to be processed. Thus, the DC and ultracentrifugation methods are hardly suitable for large-scale production of OMVs.

Size-exclusion chromatography (SEC) isolates OMVs by trapping them into nano-sized pores in porous resin particles to delay their elution time to achieve purification. SEC has frequently been employed as a secondary step to enhance the purity of crude OMV extractions in early research. Recently, however, it has been adopted for the direct isolation of OMVs from cell-free supernatants. SEC possesses the advantage of simplicity of execution and can lead to OMV isolations with high purity rates from cell-free supernatants in one step (93, 94). SEC is a gentle isolation process and maintains the native structure of OMVs better than ultracentrifugation, during which the extreme centrifugal force employed likely affects OMV structure and integrity. However, commercially available SEC columns are relatively expensive, hindering the application of SEC in OMV-based vaccine development outside purely academic fields of application.

Hydrostatic filtration dialysis (HFD) was originally developed by Musante et al. as a way to quickly identify bacterial urinary tract infections by selectively enriching urine samples (95). The method was subsequently adapted and applied to the isolation of OMVs for vaccine development in several studies (96–98). HFD has been demonstrated to be a cost-efficient, simple, and reliable way of isolating OMVs, especially in cases where large numbers of OMVs are required, such as *in vivo* immunization trials. An additional advantage of HFD is that it offers a considerably consistent reproducibility from batch to batch, a characteristic rather crucial for vaccine development (98). As HFD has only recently been developed as an OMV isolation technique, the number of studies employing HFD for OMV isolation is currently too limited to offer a proper comparison between this and other OMV isolation methods previously mentioned. Nonetheless, it remains arguably one of the most promising techniques for large-scale production of OMV vaccines.

Another technique described for the isolation of OMVs is affinity purification. This method relies on the affinity adsorption between a tag incorporated into OMVs and the ligands that specifically bind the tag. As a proof of concept, Alves et al. engineered an E. coli strain to express a recombinant OmpA protein fused with a repeated histidine sequence tag (His tag), thereby labeling the resulting OMVs secreted in culture. OMV-containing cultures were subsequently subjected to immobilized metal affinity chromatography (IMAC) to selectively isolate His-tag-marked OMVs while avoiding contamination by unlabeled wild-type OMVs (99). From the perspective of vaccine research, this method increases the consistency quality of the OMVs produced. Especially in the case of recombinant OMVs employed as delivery platforms for heterologous proteins, affinity purification simplifies the selective isolation of the loaded OMVs by the incorporation of a tag. However, IMAC purification requires direct contact between the tag and the metal ions contained in the affinity columns, limiting this technique only to OMVs loaded with outward-facing tags. OMVs loaded with heterologous proteins located in the OMV lumen cannot be isolated by affinity purification due to the inaccessibility of the tag. Furthermore, the binding capacity of the resins commonly employed in IMAC columns is limited by the relatively large size and molecular weight of the OMVs, resulting in a considerably reduced OMV recovery during affinity purification. Several strategies have been proposed to overcome the limitations imposed by the maximum binding capacity of affinity columns, including increased amounts of resin, different types of resins with a larger molecular weight cutoff, or developing novel binding materials altogether. At the moment, though, the utilization of affinity purification for the purification of OMVs remains limited to small batch sizes.

## DISCUSSION

Vaccination plays a complementary role to antibiotic treatment in reducing incidence and morbidity of bacterial infections. During the initial course of a bacterial infection, antibiotics represent an indispensable tool due to their fast mode of action and broad spectrum, which often enables clinicians to treat sudden outbreaks even when the causative agent is still uncertain (100). Infected individuals are able to clear susceptible pathogenic bacteria effectively after antibiotic administration. However, the continuous and widespread use of antibiotics has introduced a selective pressure on bacterial populations that has favored AMR bacterial strains, which again reduce the effectiveness of the same antibiotic treatment in subsequent outbreaks (101). Once the causative agent is identified, the development of effective vaccines has been shown to be an effective strategy to significantly reduce infection rates by establishing herd immunity and preventing transmission of the pathogen, consequently reducing the need for antimicrobial use and, in turn, the incidence of AMR strains (102).

First-generation vaccines represented a big step forward in both human and animal health over the past century. These vaccines are based on relatively crude formulations according to modern standards, mostly relying on the administration of inactivated or attenuated pathogenic microorganisms in order to generate immunity in healthy individuals. Despite their effectiveness, first-generation vaccines presented several safety risks, mostly due to the risk of incomplete inactivation or reversion of virulence of the pathogens included in the formulations (103). Subsequent breakthroughs in medical research revealed the immunological basis behind pathogen recognition and adaptive immunity, laying the foundation of vaccination principles as we know them today. This allowed the development of second-generation vaccines, based on subunits isolated from the pathogens rather than whole microorganisms. In subunit vaccines, only specific antigens essential for induction of protective immunity are included in the vaccine formulation, eliminating the risks associated with the administration of inactivated or attenuated vaccines (104) but increasing the reliance on adjuvants. The second generation of vaccines was also the first to benefit from advances in genome sequencing and genetic engineering technology, which culminated with the advent of reverse vaccinology as we know it (105). Reverse vaccinology enables a genome-scale *in silico* screening of antigens according to their subcellular location, conservation, and hydrophobicity to predict those that are most likely to be vaccine candidates, which greatly simplifies the process of antigen selection (106). More recently, a third generation of nucleic acid vaccines has been developed. These vaccines overcome the need to isolate or synthetize the antigens needed for immunization, relying instead on having them expressed directly by the host cells (107). Fourth-generation vaccines, also referred to as next-generation vaccines, have an additional advantage as they can be developed as long as the sequence information of the pathogen is known (108). Examples include APC-targeting vaccines, nucleic acid vaccines delivered by nanoparticles, and artificial APC vaccines. APC-targeting vaccines can elicit particular types of responses needed for clearing specific pathogens by targeting different receptors of the APCs (109). Nucleic acid vaccines delivered by nanoparticles, mostly lipid nanoparticles, can stabilize nucleic acids, particularly mRNA, to enable the entry into cells and induce both humoral and cellular immune responses (108). Artificial APCs mimic APCs attached with epitope-loaded MHC molecules and costimulatory molecules, bypassing antigen processing and directly activating T cells to generate large numbers of immune effectors and achieve faster clearance of pathogens (110).

Due to their nonreplicative nature, antigenic properties, and adjuvanticity, OMVs as immunogens do not really fit in any of the previously mentioned vaccine categories. As such, OMV-based vaccines constitute a category of their own.

The pluripotency of OMVs as adjuvants, antigens, and delivery platforms is being extensively investigated and has been proven by the development of several OMV-based pharmaceutical prototypes in the last 2 decades, which have been well summarized in other reviews (44, 111, 112). Nonetheless, releasing the full potential of OMVs will require additional efforts focused on the current limitations in production and characterization of these otherwise versatile tools. For instance, balancing essential factors, such as high immunogenicity and low toxicity, optimal antigenic profile, and cost-effective production, must to be taken into account when formulating OMV-based pharmaceuticals. This is particularly required when the intended use is aimed at humans in low-income countries and the food production sector where resources are scare and profit margins are small.

Despite the universal nature of OMV secretion among Gram-negative bacterial species, secreted OMVs are often a rather heterogeneous mix of particles possessing significantly different attributes and functions. As a consequence of this intrinsic variability, OMVs isolated from different bacterial species have been shown to present very different protein and, consequently, immunological profiles. For this reason, very limited assumptions can be made at the beginning of any research endeavor focusing on previously undescribed OMVs. Any novel OMV-producing strains need to be individually characterized and often genetically engineered to increase OMV yields, a lengthy and time-consuming process. Further insights into the relationship between OMV composition and their resulting immunological profile would also be beneficial for accelerating the development of novel OMV products.

In conclusion, the immunological properties of OMVs characterize them as a promising new avenue for the development of novel vaccine formulations and will almost certainly represent a useful tool at our disposal in combating bacterial infectious diseases and tackling the global challenge posed by AMR.
