# Supplementary material for: Bacterial Outer Membrane Vesicles as a Versatile Tool in Vaccine Research and the Fight against Antimicrobial Resistance
Source: mBio. 2021 Aug 10;12(4):e01707-21. doi: 10.1128/mBio.01707-21 (PMC8406158; doi:10.1128/mBio.01707-21)
Supplement: TABLE S1 [file mbio.01707-21-st001.docx]

Table S1. Examples of experimental OMVs-based vaccines

| **Species** | **Vaccine formulation** | **Animal model** | **Survival rate*** | | **References** |
| --- | --- | --- | --- | --- | --- |
| ***Human pathogens*** | | | | | |
| *Acinetobacter baumannii* | Native OMVs | Mouse | | 73.3% | [1] |
| *Vibrio cholerae* | Native OMVs | Mouse | | NA | [2] |
| *Bordetella pertussis* | Native OMVs | Mouse | | NA | [3] |
| *Pseudomonas aeruginosa* | Native OMVs | Mouse | | 80%** | [4] |
| ***Animal pathogens*** | | | | | |
| Avian pathogenic *Escherichia coli* | Native OMVs | Chicken | | 100% | [5] |
| *Haemophilus parasuis* | Native OMVs | Pig | | 100% | [6] |
| *Gallibacterium anatis* | Native OMVs combined with recombinant proteins | Chicken | | NA | [7] |
| *Actinobacillus pleuropneumoniae* | Native OMVs combined with recombinant proteins | Pig | | NA | [8] |
| ***Zoonotic pathogens*** |  |  | |  |  |
| *Campylobacter jejuni* | Native OMVs enriched with CjaA protein | Chicken | | NA | [9] |
| *Brucella melitensis* | Native OMVs | Mouse | | NA | [10] |
| *Salmonella Typhimurium* | Native OMVs | Mouse | | 100% | [11] |
| *Pasteurella multocida* | Native OMVs | Mouse | | NA | [12] |

* Survival rate shows animals survived after challenge experiments. NA indicates that no challenge experiments performed or no data on survival rate are available.

** Result from passive immunization assays using hyperimmune sera from mice immunized with OMVs.

1. Huang W, Yao Y, Long Q, Yang X, Sun W, Liu C, Jin X, Li Y, Chu X, Chen B, Ma Y (2014) Immunization against multidrug-resistant Acinetobacter baumannii effectively protects mice in both pneumonia and sepsis models. PLoS One 9 (6):e100727. doi:10.1371/journal.pone.0100727

2. Schild S, Nelson EJ, Camilli A (2008) Immunization with Vibrio cholerae outer membrane vesicles induces protective immunity in mice. Infection and immunity 76 (10):4554-4563. doi:10.1128/IAI.00532-08

3. Raeven RHM, Rockx-Brouwer D, Kanojia G, van der Maas L, Bindels THE, Ten Have R, van Riet E, Metz B, Kersten GFA (2020) Intranasal immunization with outer membrane vesicle pertussis vaccine confers broad protection through mucosal IgA and Th17 responses. Scientific reports 10 (1):7396. doi:10.1038/s41598-020-63998-2

4. Zhang X, Yang F, Zou J, Wu W, Jing H, Gou Q, Li H, Gu J, Zou Q, Zhang J (2018) Immunization with Pseudomonas aeruginosa outer membrane vesicles stimulates protective immunity in mice. Vaccine 36 (8):1047-1054. doi:10.1016/j.vaccine.2018.01.034

5. Wang H, Liang K, Kong Q, Liu Q (2019) Immunization with outer membrane vesicles of avian pathogenic Escherichia coli O78 induces protective immunity in chickens. Vet Microbiol 236:108367. doi:10.1016/j.vetmic.2019.07.019

6. McCaig WD, Loving CL, Hughes HR, Brockmeier SL (2016) Characterization and Vaccine Potential of Outer Membrane Vesicles Produced by Haemophilus parasuis. PLoS One 11 (3):e0149132. doi:10.1371/journal.pone.0149132

7. Antenucci F, Arak H, Gao J, Allahgadry T, Thofner I, Bojesen AM (2020) Hydrostatic Filtration Enables Large-Scale Production of Outer Membrane Vesicles That Effectively Protect Chickens against Gallibacterium anatis. Vaccines (Basel) 8 (1). doi:10.3390/vaccines8010040

8. Antenucci F, Fougeroux C, Deeney A, Orskov C, Rycroft A, Holst PJ, Bojesen AM (2018) In vivo testing of novel vaccine prototypes against Actinobacillus pleuropneumoniae. Veterinary research 49 (1):4. doi:10.1186/s13567-017-0502-x

9. Godlewska R, Kuczkowski M, Wyszynska A, Klim J, Derlatka K, Wozniak-Biel A, Jagusztyn-Krynicka EK (2016) Evaluation of a protective effect of in ovo delivered Campylobacter jejuni OMVs. Appl Microbiol Biotechnol 100 (20):8855-8864. doi:10.1007/s00253-016-7699-x

10. Bagheri Nejad R, Yahyaraeyat R, Es-Haghi A, Nayeri Fasaei B, Zahraei Salehi T (2019) Induction of specific cell-mediated immune responses and protection in BALB/c mice by vaccination with outer membrane vesicles from a Brucella melitensis human isolate. APMIS : acta pathologica, microbiologica, et immunologica Scandinavica 127 (12):797-804. doi:10.1111/apm.12997

11. Liu Q, Liu Q, Yi J, Liang K, Liu T, Roland KL, Jiang Y, Kong Q (2016) Outer membrane vesicles derived from Salmonella Typhimurium mutants with truncated LPS induce cross-protective immune responses against infection of Salmonella enterica serovars in the mouse model. International journal of medical microbiology : IJMM 306 (8):697-706. doi:10.1016/j.ijmm.2016.08.004

12. Roier S, Fenninger JC, Leitner DR, Rechberger GN, Reidl J, Schild S (2013) Immunogenicity of Pasteurella multocida and Mannheimia haemolytica outer membrane vesicles. International journal of medical microbiology : IJMM 303 (5):247-256. doi:10.1016/j.ijmm.2013.05.001
