# Supplementary material for: Bacterial Outer Membrane Vesicles as a Versatile Tool in Vaccine Research and the Fight against Antimicrobial Resistance
Source: mBio. 2021 Aug 10;12(4):e01707-21. doi: 10.1128/mBio.01707-21 (PMC8406158; doi:10.1128/mBio.01707-21)
Supplement: TABLE S2 [file mbio.01707-21-st002.docx]

Table S2. Methods for increasing OMV yields

| **Methods** | **Examples** | **Resulting OMV types*** | **References** |
| --- | --- | --- | --- |
| **Physical methods** |  |  |  |
| sonication | *Haemophilus parasuis* | nOMVs | [1] |
| temperature stress | *Pseudomonas putida* | nOMVs | [2] |
| **Chemical methods** |  |  |  |
| detergents | *Neisseria meningitidis* | dOMVs | [3] |
| chelators | *Neisseria meningitidis* | nOMVs | [4] |
| Hydrogen peroxide | *Pseudomonas aeruginosa* | nOMVS | [5] |
| **Genetic engineering methods** |  |  |  |
| Tol-Pal system | *Escherichia coli* | sOMVs | [6] |
| *nlpI* | *Actinobacillus pleuropneumoniae* | sOMVs | [7-9] |
| *degS* | *Salmonella enterica* | sOMVs | [8,10] |

*To keep consistent with the literature, OMVs are classified using the nomenclature reported in the corresponding study [11]. nOMVs: native OMVs extracted from cells with detergent-free methods; dOMVs, OMVs extracted from cells with detergent; sOMVs, OMVs spontaneously released by cells.

1. McCaig WD, Loving CL, Hughes HR, Brockmeier SL (2016) Characterization and Vaccine Potential of Outer Membrane Vesicles Produced by Haemophilus parasuis. PLoS One 11 (3):e0149132. doi:10.1371/journal.pone.0149132

2. Baumgarten T, Sperling S, Seifert J, von Bergen M, Steiniger F, Wick LY, Heipieper HJ (2012) Membrane vesicle formation as a multiple-stress response mechanism enhances Pseudomonas putida DOT-T1E cell surface hydrophobicity and biofilm formation. Applied and environmental microbiology 78 (17):6217-6224. doi:10.1128/aem.01525-12

3. Holst J, Martin D, Arnold R, Huergo CC, Oster P, O’Hallahan J, Rosenqvist E (2009) Properties and clinical performance of vaccines containing outer membrane vesicles from Neisseria meningitidis. Vaccine 27 (2):B3-12

4. van de Waterbeemd B, Streefland M, van der Ley P, Zomer B, van Dijken H, Martens D, Wijffels R, van der Pol L (2010) Improved OMV vaccine against Neisseria meningitidis using genetically engineered strains and a detergent-free purification process. Vaccine 28 (30):4810-4816. doi:10.1016/j.vaccine.2010.04.082

5. Macdonald IA, Kuehn MJ (2013) Stress-induced outer membrane vesicle production by Pseudomonas aeruginosa. Journal of bacteriology 195 (13):2971-2981. doi:10.1128/JB.02267-12

6. Bernadac A, Gavioli M, Lazzaroni JC, Raina S, Lloubès R (1998) Escherichia coli tol-pal mutants form outer membrane vesicles. Journal of bacteriology 180 (180):4872-4878

7. Schwechheimer C, Rodriguez DL, Kuehn MJ (2015) NlpI-mediated modulation of outer membrane vesicle production through peptidoglycan dynamics in Escherichia coli. MicrobiologyOpen 4 (3):375-389. doi:10.1002/mbo3.244

8. Antenucci F, Fougeroux C, Bosse JT, Magnowska Z, Roesch C, Langford P, Holst PJ, Bojesen AM (2017) Identification and characterization of serovar-independent immunogens in Actinobacillus pleuropneumoniae. Veterinary research 48 (1):74. doi:10.1186/s13567-017-0479-5

9. Ojima Y, Sawabe T, Konami K, Azuma M (2019) Construction of hypervesiculation Escherichia coli strains and application for secretory protein production. Biotechnol Bioeng. doi:10.1002/bit.27239

10. Nevermann J, Silva A, Otero C, Oyarzun DP, Barrera B, Gil F, Calderon IL, Fuentes JA (2019) Identification of Genes Involved in Biogenesis of Outer Membrane Vesicles (OMVs) in Salmonella enterica Serovar Typhi. Frontiers in microbiology 10:104. doi:10.3389/fmicb.2019.00104

11. van der Pol L, Stork M, van der Ley P (2015) Outer membrane vesicles as platform vaccine technology. Biotechnology Journal 10 (11):1689-1706. doi:10.1002/biot.201400395
